# Supplementary material for: ‘Mix-it-up’: accessible time-resolved cryo-EM on the millisecond timescale
Source: IUCrJ. 2025 Oct 27;12(Pt 6):710–24. doi: 10.1107/S2052252525008838 (PMC12573921; doi:10.1107/S2052252525008838)
Supplement: Supplementary file 1 [file m-12-00710-sup1.pdf]

IUCrJ

Volume 12 (2025)

Supporting information for article:

"Mix-it-up": accessible time-resolved cryo-EM on the millisecond timescale

Lauren Alexandrescu, William Lessin and Gabriel C. Lander

Table S1 Cryo-EM data collection and image analysis.

	Apo-ferritin	Aldolase	CCMV contracted	CCMV expanded	CCMV pH shift	GroEL barrel, no ATP	GroEL barrel, 100 ms	GroEL barrel, 300 ms	GroEL bullet, 300 ms	GroEL football, 300 ms	GroEL barrel, 700 ms	GroEL bullet, 700 ms	GroEL football, 700 ms
EMDB ID	70747	70748	70749	70750	70751	70752	70759	70753	70754	70755	70756	70757	70758
**Data collection**													
Microscope	FEI Talos Arctica		FEI Talos Arctica			FEI Talos Arctica		FEI Talos Arctica			FEI Talos Arctica		
Camera	TFS Falcon 4i		TFS Falcon 4i			TFS Falcon 4i		TFS Falcon 4i			TFS Falcon 4i		
Magnification (nominal / at detector)	150,000/148,936		150,000/148,936			150,000/148936		150,000/148936			150,000/148936		
Voltage (kV)	200		200			200		200			200		
Data acquisition software	EPU v3.9.1.8206		EPU v3.9.1.8206			EPU v3.9.1.8206		EPU v3.9.1.8206			EPU v3.9.1.8206		
Exposure navigation	Image shift to 8 µm		Image shift to 8 µm			Image shift to 8 µm		Image shift to 8 µm			Image shift to 8 µm		
Total electron exposure (e^-/Å^2)	50		50			50		50			50		
Exposure rate (e-/pixel/sec)	10.53		10.75	9.93	10.90	10.90	10.94	9.66			10.90		
Total exposure time (s)	4.2		4.1	4.5	4.1	4.1	4.1	4.6			4.1		
Number of frames	1,296		1,008	1,377	1,251	1,251	1,251	1,395			1,251		
Pixel size (Å) per pixel	0.94		0.94			0.94		0.94			0.94		
Defocus range (µm)	-1 to -1.4		-1 to -1.4			-1 to -1.4		-1 to -1.4			-1 to -1.4		
Micrographs collected (no.)	2,476		292	595	6,007	601	2,645	5,301			6,063		
**Data analysis**													
Movie fractions (no.)	40		40	40	40	40	40	40			40		
Total extracted picks (no.)	1,701,352	820,058	7,994	62,017	68,810	60,116	119,235	255,389			118,543		
Refined particles (no.)	81,562	185,420	5,397	1,885	6,952	11,859	11,931	14,813	25,588	7,696	3,605	8,753	2,121
Final particles (no.)	79,193	185,420	5,369	1,665	6,889	11,777	11,864	14,730	25,430	7,653	3,588	8,711	2,111
Symmetry	O	D2	I	I	I	D7	D7	D7	C7	D7	D7	C7	D7
Global Resolution (Å)													
FSC 0.143 (no mask / mask)	2.8 / 2.5	3.2 / 2.9	3.8 / 3.2	8.4 / 6.9	4.6 / 3.9	3.8 / 3.2	3.9 / 3.4	4.2 / 3.6	3.9 / 3.3	6.8 / 4.8	7.0 / 4.7	4.4 / 3.5	8.1 / 7.1
Local resolution range (Å)	2.1–8.8	2.5–38.1	2.0–49.5	1.9–17.6	2.0–61.5	2.0-31.8	3.1–41.5	4.3–38.2	1.9-17.4	5.0–51.4	2.0-70.5	2.0–55.3	1.9-17.4
cFSCs cFAR	0.95	0.64	0.97	0.85	0.98	0.80	0.65	0.69	0.79	0.31	0.31	0.79	0.42
Map sharpening B-factor (Å^2)	-97.1	-121.2	-79.8	-383.1	-82.8	-75.4	-75.2	-84.7	-74.3	-180.9	-118.4	-60.5	-685.9

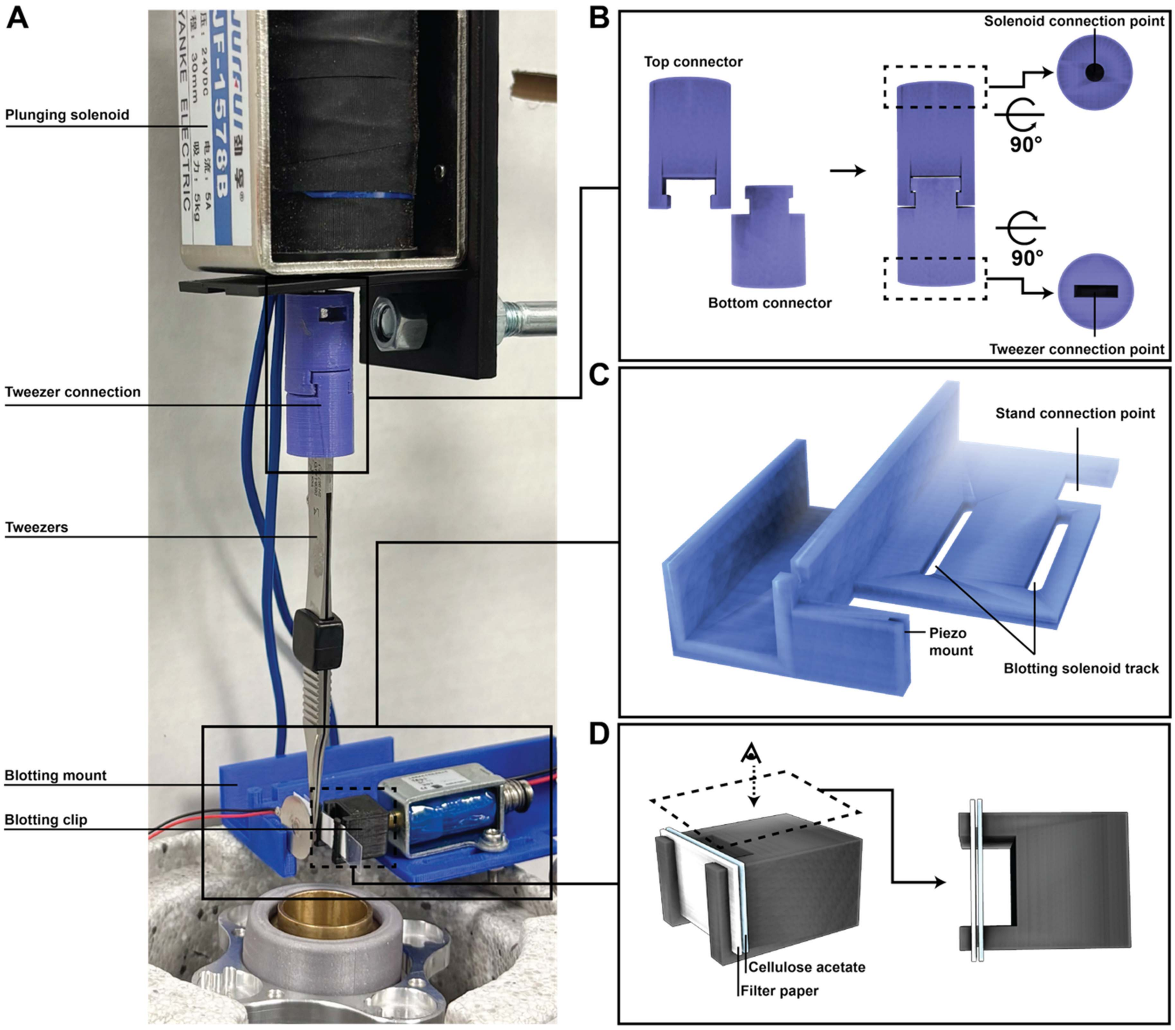

Figure S1 Modifications implemented in “Mix-it-up”. **A)** Photograph of the Mix-it-Up device denoting modifications introduced. **B)** Schematic of the updated tweezer connection design, which consists of a top component that connects to the plunging solenoid and a bottom component that connects to the tweezers. The components are shown disjoined (left) and joined (right). The top and bottom views show the connection points to the solenoid and tweezers, respectively. **C)** Schematic of the blotting mount. **D)** Side (left) and top (right) views of the blotting clip with filter paper positioned between the two pillars and a strip of cellulose acetate used as backing support.

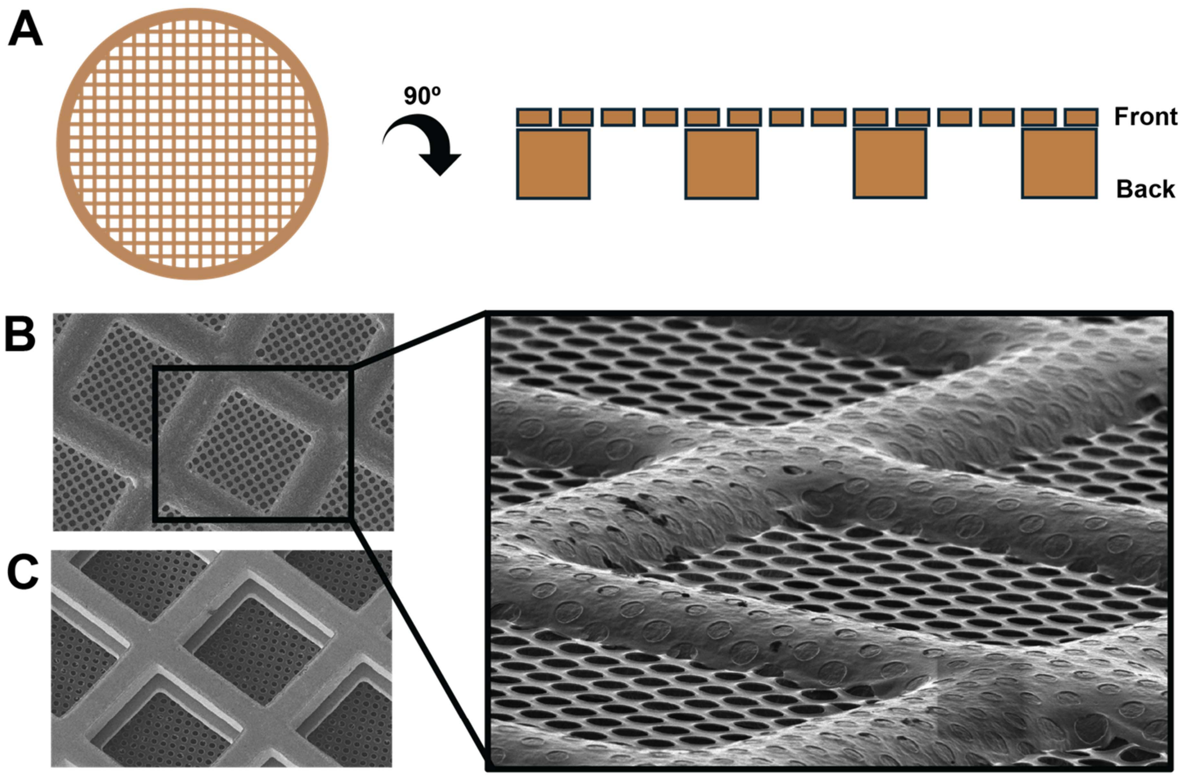

Figure S2 EM grid architecture. **A)** 2D schematic of an EM grid (left) and a representative schematic of the side view of several squares depicting the holey foil (front) and grid bars (back). **B)** Scanning electron microscopy (SEM) image of the front of a grid, showing a rounded surface of the grid bars overlaid with a holey foil (inset). **C)** SEM image of the back of a grid.

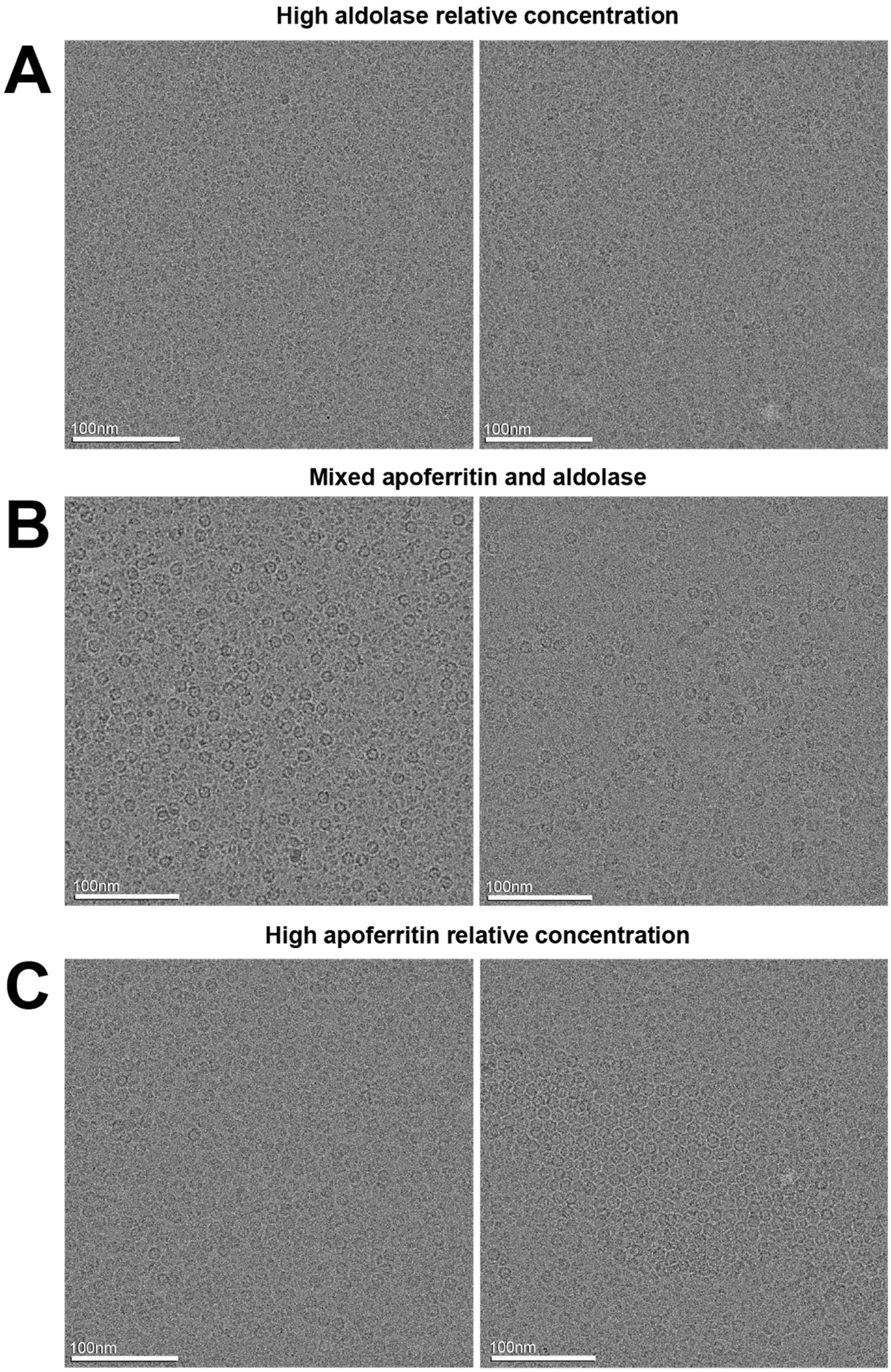

Figure S3 Aldolase and apoferritin particle distribution in mixed sample prepared with MIU. Representative micrographs from grids prepared with: **(A)** high aldolase concentration, **(B)** mixed apoferritin and aldolase concentrations, and **(C)** high apoferritin concentrations**.**

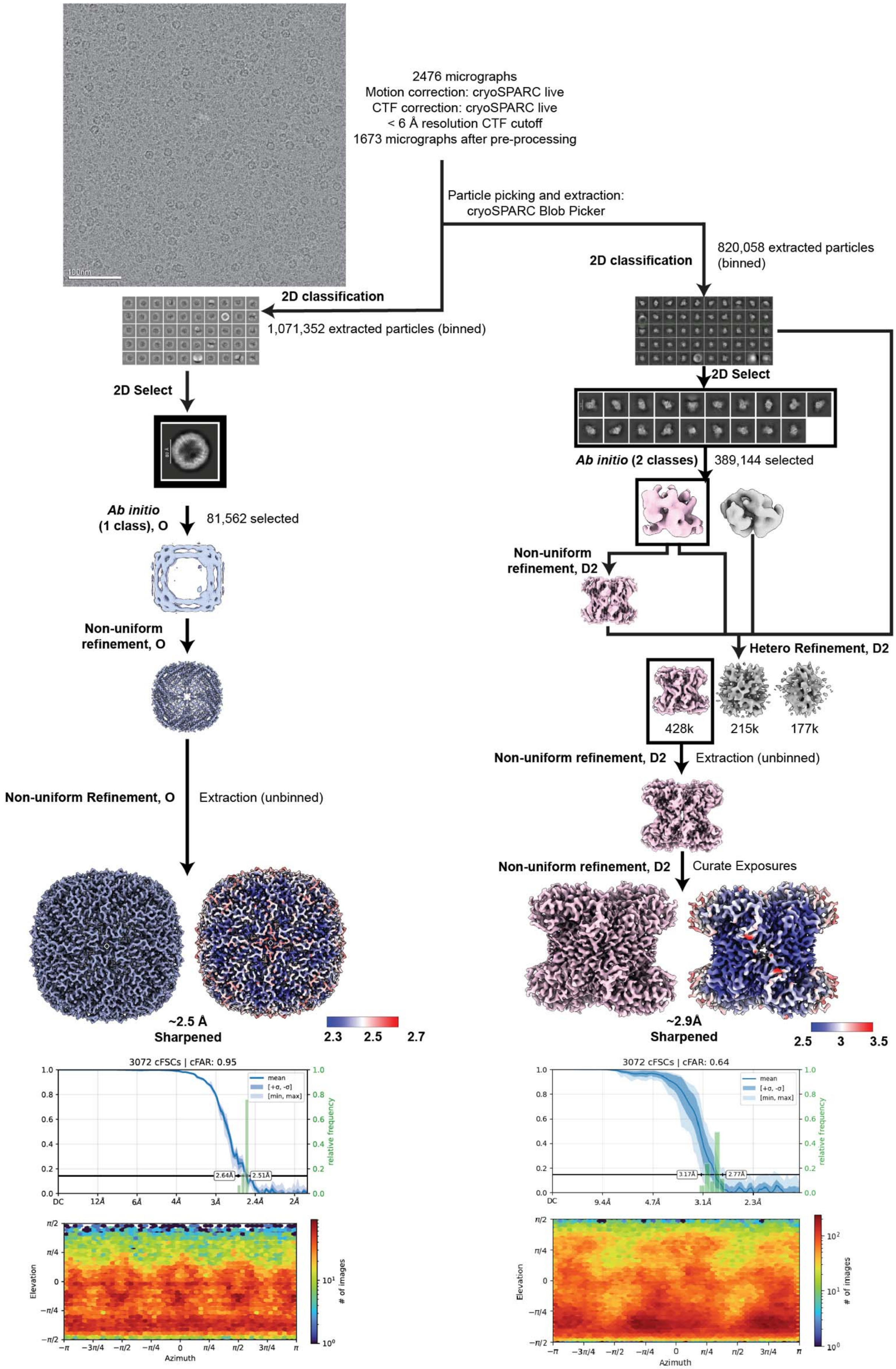

Figure S4 Processing pipeline of the mixed apoferritin and aldolase dataset prepared with MIU. Representative processing pipeline for mixed sample of apoferritin and aldolase. Motion and CTF correction were performed in

cryoSPARC Live, as well as picking and extraction of apoferritin particles. Micrographs were exported to cryoSPARC for aldolase particle picking and extraction. Following 2D classification of apoferritin particles, a single apoferritin class was selected. An *ab initio* reconstruction was generated with octahedral symmetry imposed, and the resulting reconstruction was further refined. Aldolase particles were processed in a similar manner with the following exceptions: a single subset of particles was generated of the best class averages following 2D classification. Following *ab initio* reconstruction, initial refinement, and heterogenous refinement, the particles were further refined, and exposures were curated to further filter out poor quality particles. For apoferritin and aldolase, final refinement of the selected particles yielded maps at ~2.5 Å and ~2.9 Å resolution, respectively. Final maps are shown next to a local resolution map. The conical FSC plot overlaid with a resolution histogram and corresponding angular distribution plot are shown below the final maps.

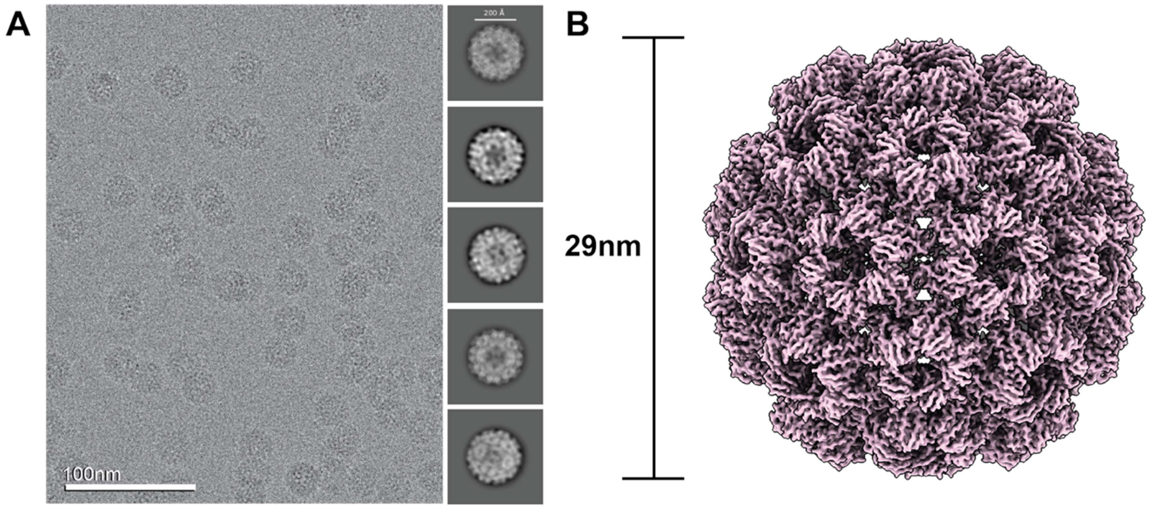

Figure S5 Control, contracted CCMV. **A)** Representative micrograph and 2D class averages of CCMV sample in pH 4.6 buffer. **B)** EM density map of the contracted state exhibiting a 29 nm diameter.

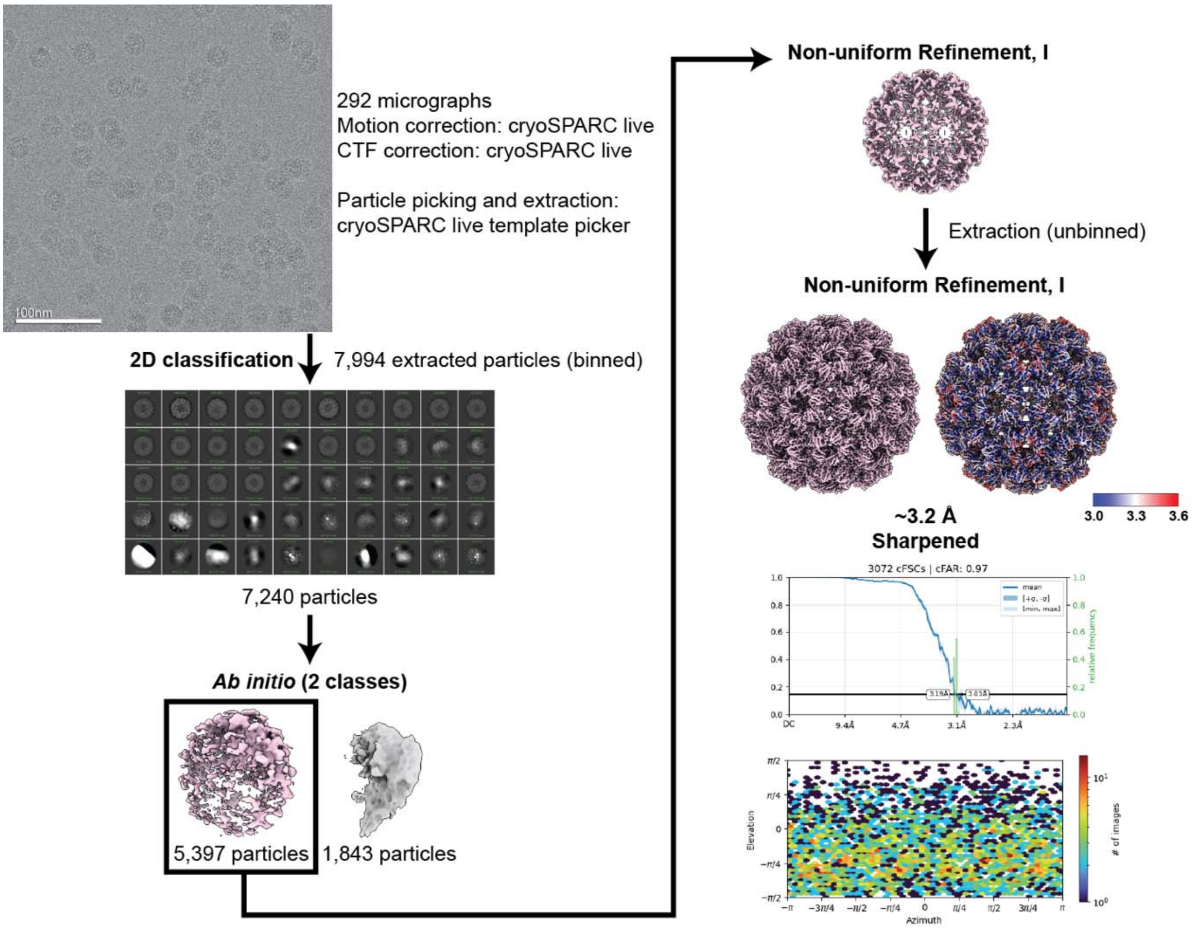

Figure S6 Processing pipeline of the control, contracted CCMV state dataset. Representative processing pipeline for CCMV dataset. Motion and CTF correction, initial particle picking, and initial 2D classification were performed in cryoSPARC Live. Template-based picking was used to refine particle selection, followed by additional 2D classification and *ab initio* reconstruction. Particles from the best class were used for 3D refinement with imposed icosahedral symmetry. After re-extraction, particles were further refined to yield a final map at ~3.2 Å resolution. The final map is shown next to a local resolution map. A conical FSC plot overlaid with a histogram of resolutions and an angular distribution plot are shown below the final maps.

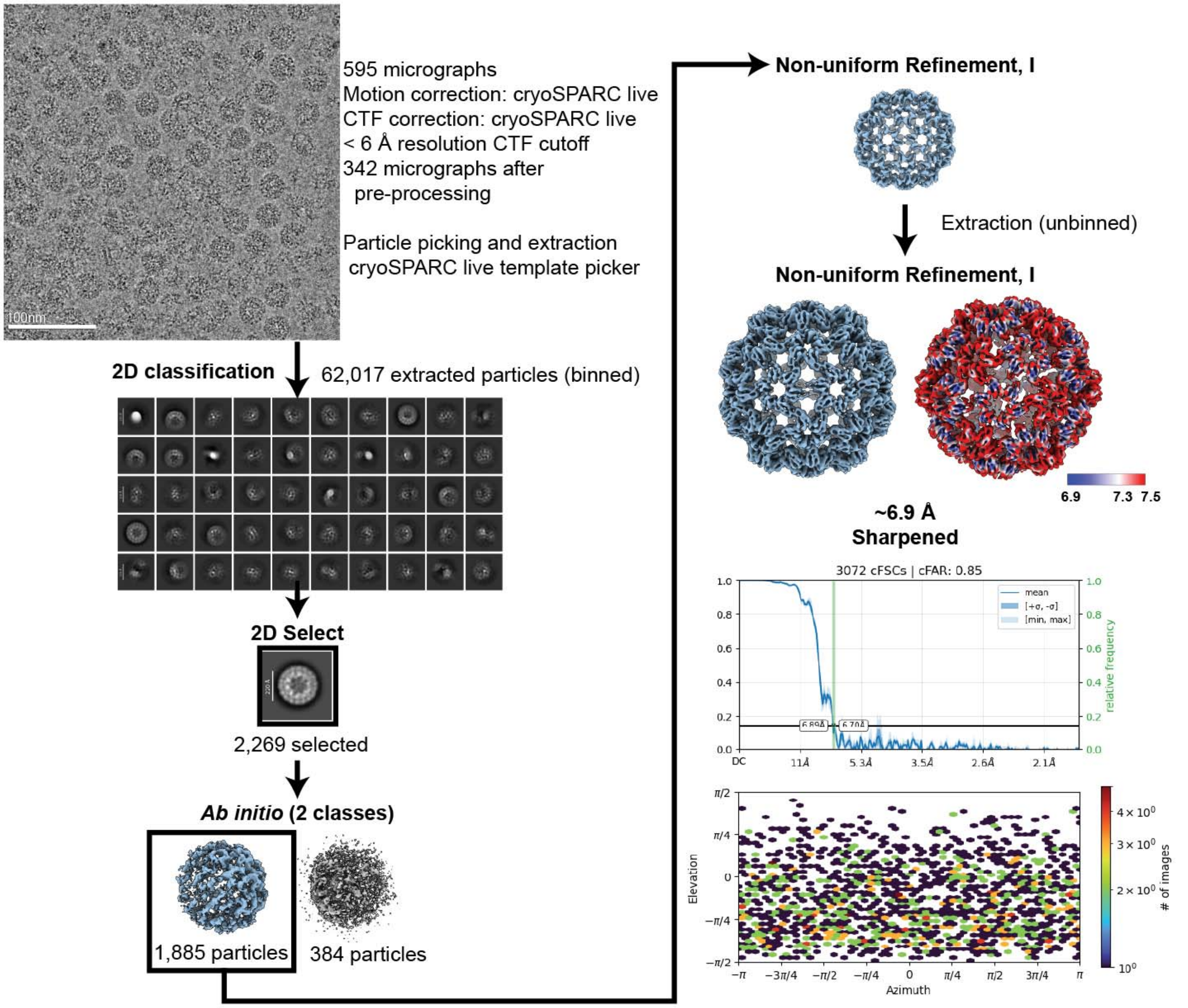

Figure S7 Processing pipeline of the control, expanded CCMV state dataset. Representative processing pipeline for the expanded CCMV dataset. Motion and CTF correction were performed in cryoSPARC Live, with a resolution cutoff applied during preprocessing. Initial blob-based particle picking was followed by template-based picking and 2D classification. A selected subset of particles was used for *ab initio* reconstruction and 3D refinement was performed with imposed icosahedral symmetry. After re-extraction, particles were further refined to yield a final map at ~6.9 Å resolution. The final map is shown next to a local resolution map. The conical FSC plot overlaid with a resolution histogram and corresponding angular distribution plot are shown below the final map.

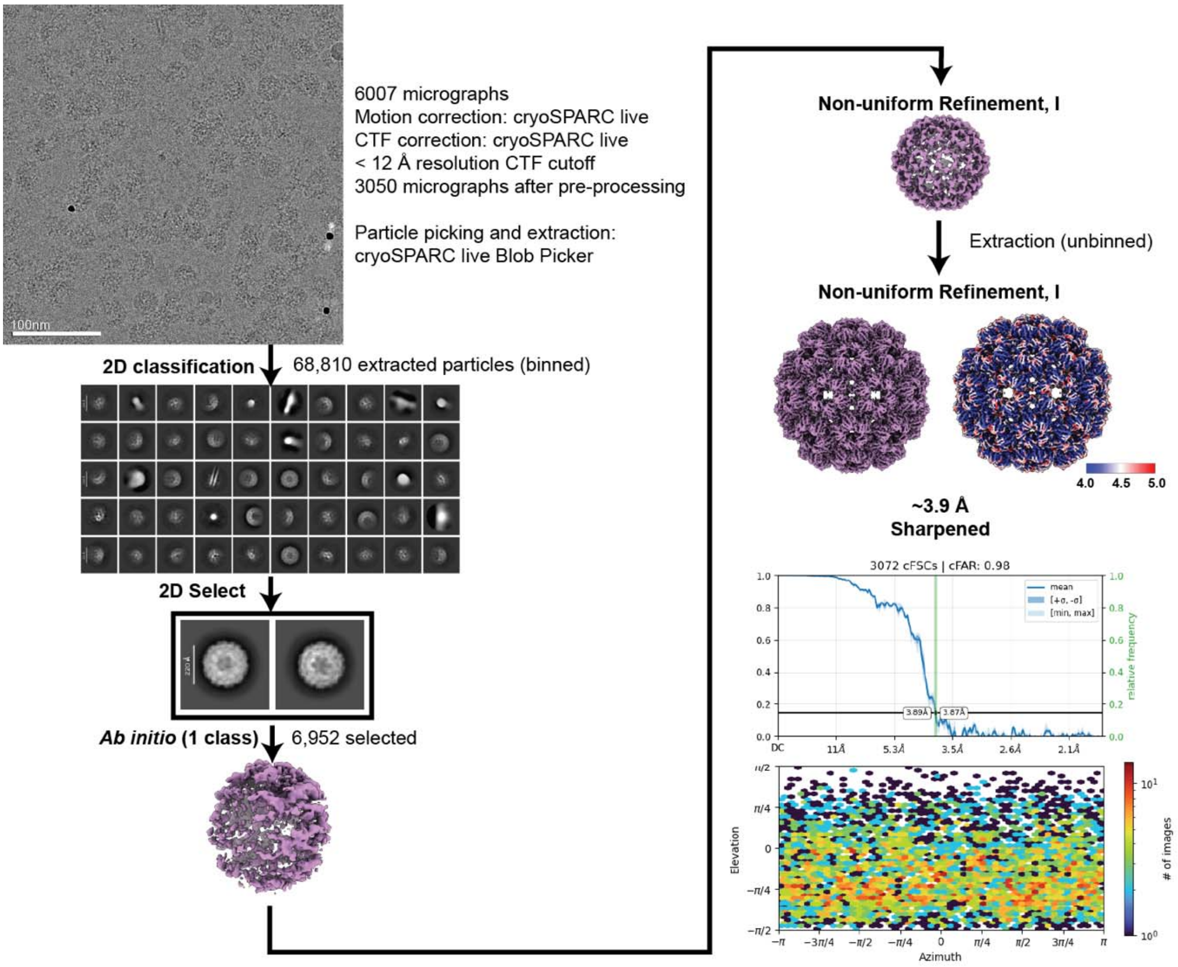

Figure S8 Processing pipeline of the pH-shift CCMV dataset prepared with MIU. Representative processing pipeline for the pH-shift CCMV dataset. Motion and CTF correction were performed in cryoSPARC Live. Particles were picked using blob-based picking, followed by 2D classification and selection of a particle subset for *ab initio* reconstruction. The resulting volume was refined with icosahedral symmetry imposed. After re-extraction, particles were further refined to yield a final map at ~3.9 Å resolution. The final map is shown next to a local resolution map. The conical FSC plot overlaid with a resolution histogram and corresponding angular distribution plot are shown below the final map.

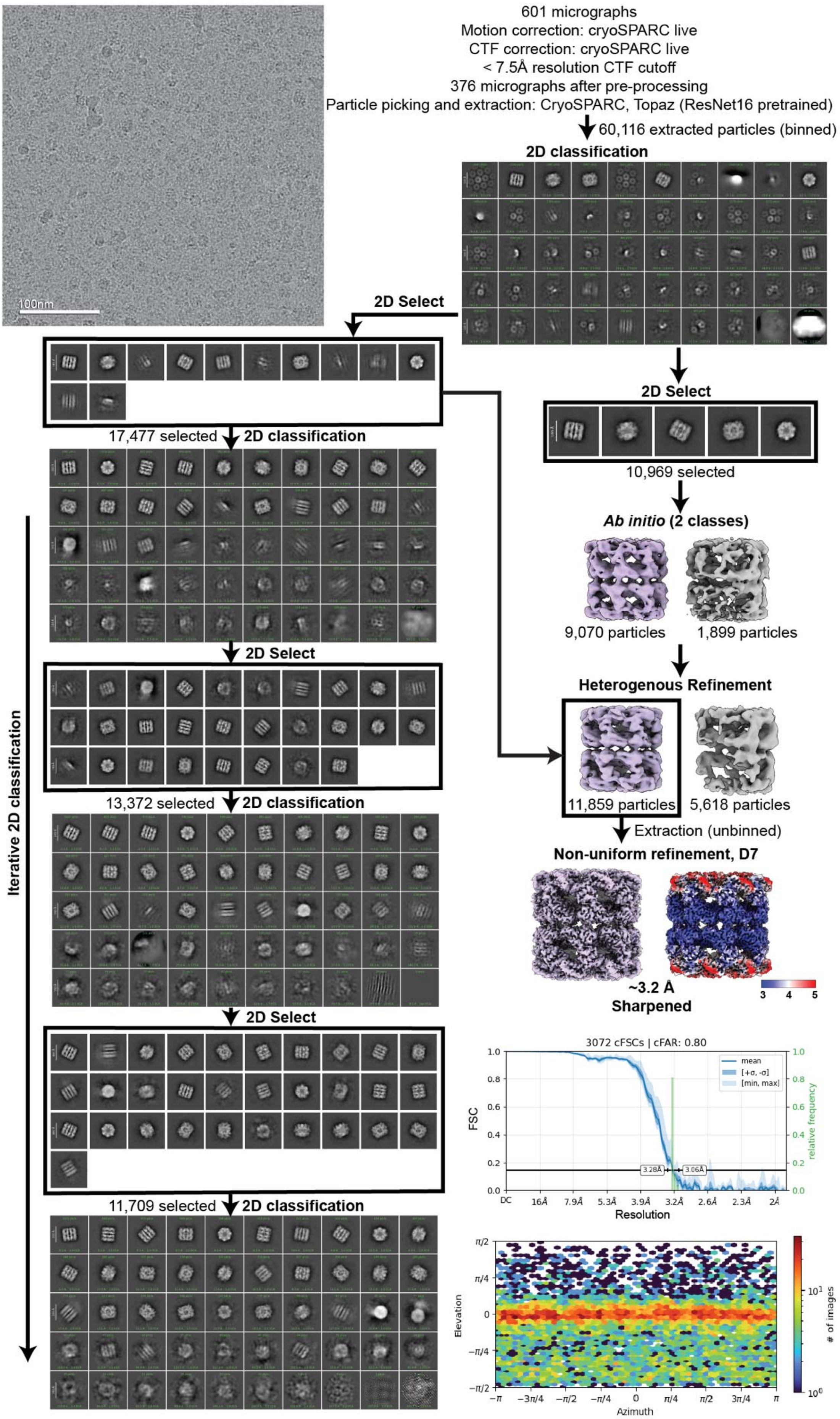

Figure S9 Processing pipeline of the control dataset of GroEL in the presence of GroES and absence of ATP. Representative processing pipeline for the GroEL + GroES control dataset prepared in the absence of ATP. Motion

and CTF correction were performed in cryoSPARC Live. Micrographs were exported to cryoSPARC, and particles were picked using Topaz with a pretrained model, followed by initial 2D classification. Iterative 2D classification was also performed to confirm the absence of GroEL/ES complexes. A subset of particles resembling GroEL was selected for *ab initio* reconstruction, and the resulting volumes were further refined in a heterogeneous refinement job using a broader particle set. High-quality particles were then re-extracted and refined with imposed D7 symmetry to yield a final map at ~3.2 Å resolution. The conical FSC plot overlaid with a resolution histogram and corresponding angular distribution plot are shown below the final map.

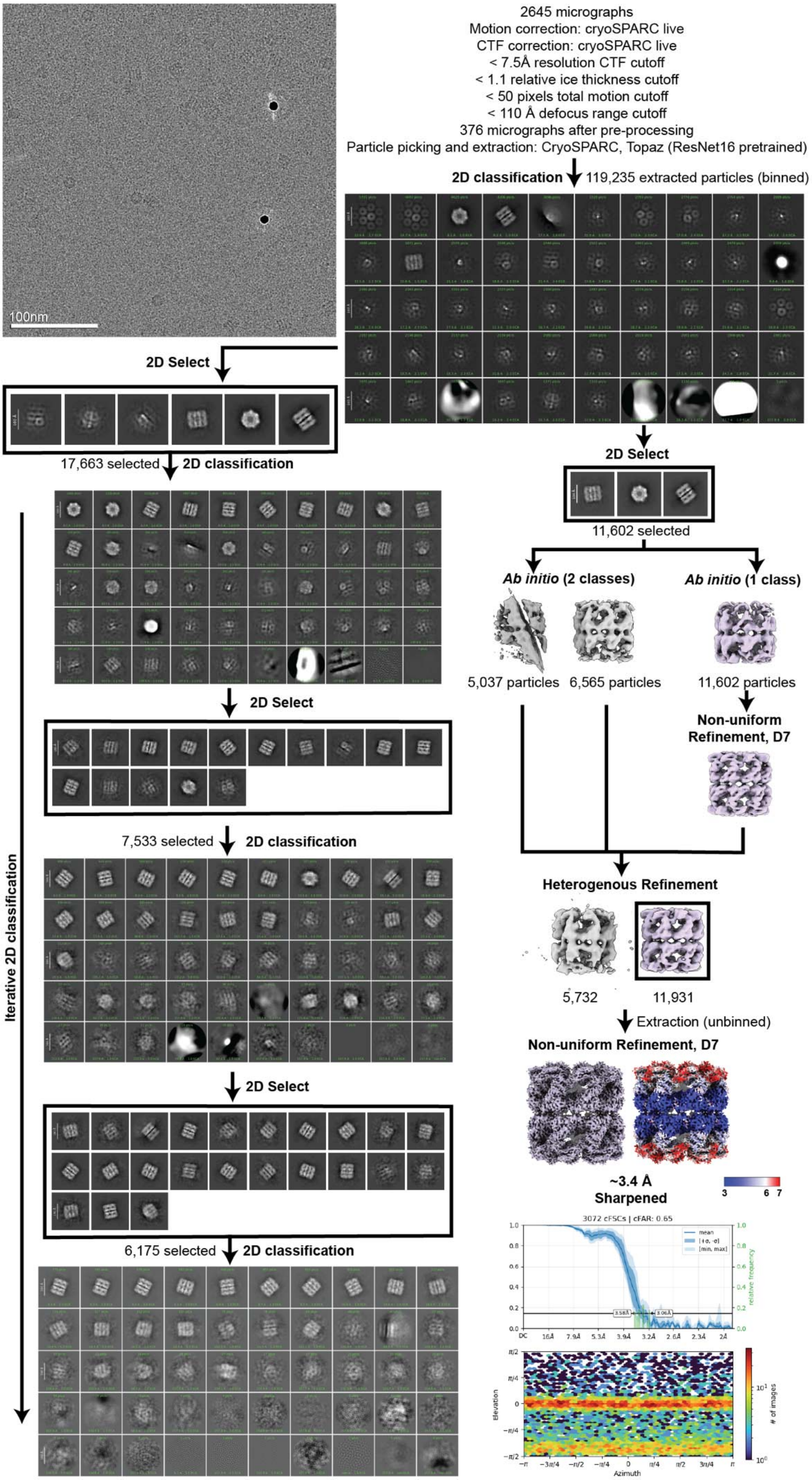

Figure S10 Processing pipeline of the 100 ms GroEL/ES + ATP dataset. Representative processing pipeline for the 100 ms GroEL + GroES + ATP dataset. Motion and CTF correction were performed in cryoSPARC Live.

Micrographs were exported to cryoSPARC, and particles were picked using Topaz with a pretrained model, followed by 2D classification and selection of GroEL particles, resembling the “barrel” (uncapped) conformational state. Iterative 2D classification was performed in parallel to confirm the absence of additional conformational states. *Ab initio* reconstructions were generated and the “best” class was further refined with symmetry imposed. Heterogeneous refinement was then used to sort high-quality particles from a broader subset selection. The best particles were re-extracted and refined with D7 symmetry to yield a final map at ~3.4 Å resolution. The final map is shown next to a local resolution map. The conical FSC plot overlaid with a resolution histogram and corresponding angular distribution plot are shown below the final maps.

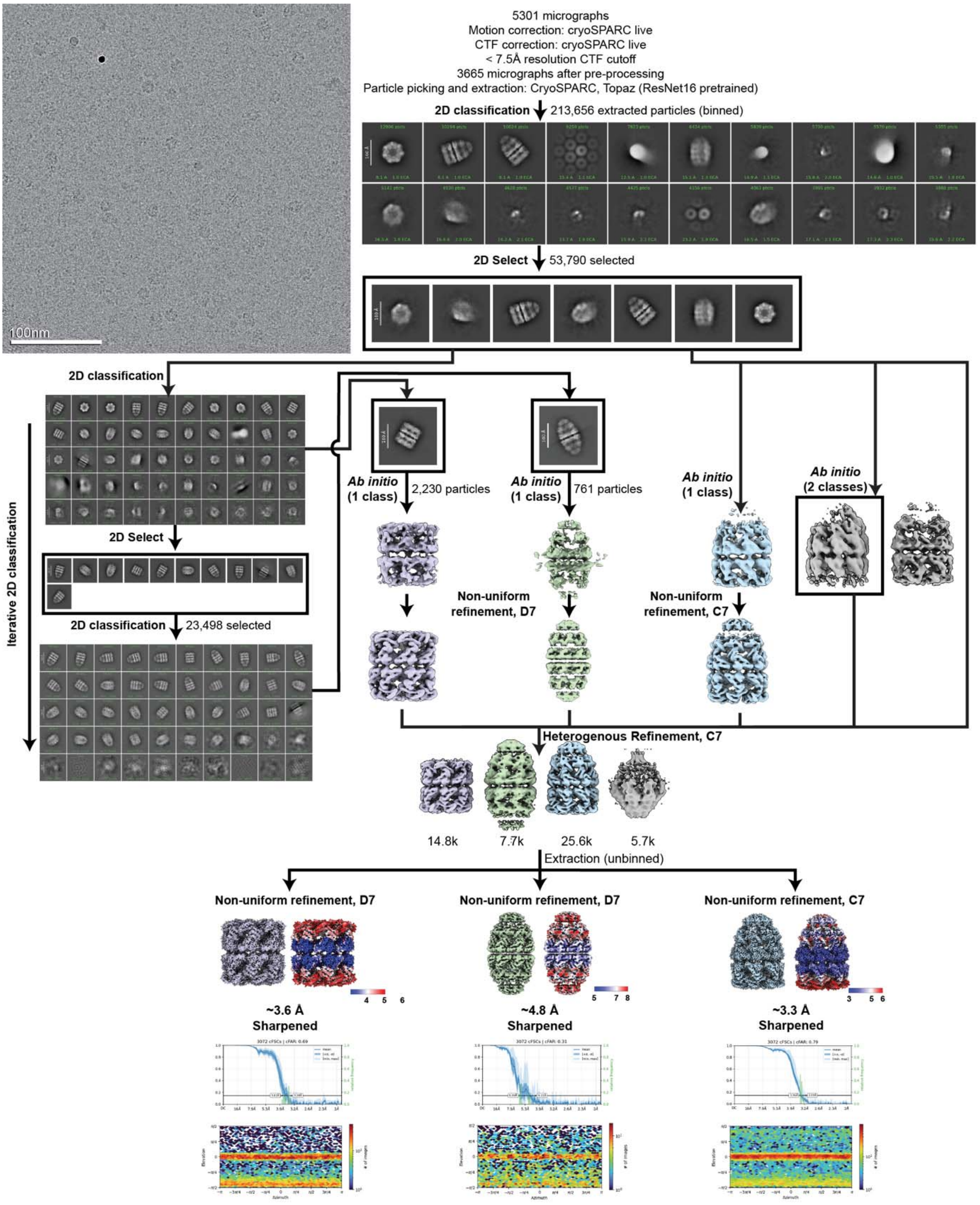

Figure S11 Processing pipeline of the 300 ms GroEL/ES + ATP dataset. Representative processing pipeline for the 300 ms GroEL+ GroES + ATP dataset. Motion and CTF correction were performed in cryoSPARC Live. Micrographs were exported to cryoSPARC, and particles were picked using Topaz with a pretrained model, followed by initial 2D classification and selection of GroEL/ES complexes, all resembling the “bullet” (single-capped) conformation. Further rounds of 2D classification revealed two additional conformational states – the barrel (uncapped), and football (double-capped). *Ab initio* reconstructions were performed independently for each of the three conformational states. These reconstructions, along with a junk volume, were used in heterogeneous refinement with C7 symmetry imposed to further refine particle sets. Sorted particles were re-extracted and refined with symmetry to yield final maps at ~3.3 Å (bullet), ~3.6 Å (barrel), and ~4.8 Å (football) resolution. Final maps are shown next to

local resolution maps. The conical FSC plot overlaid with a resolution histogram and corresponding angular distribution plot are shown below the final maps.

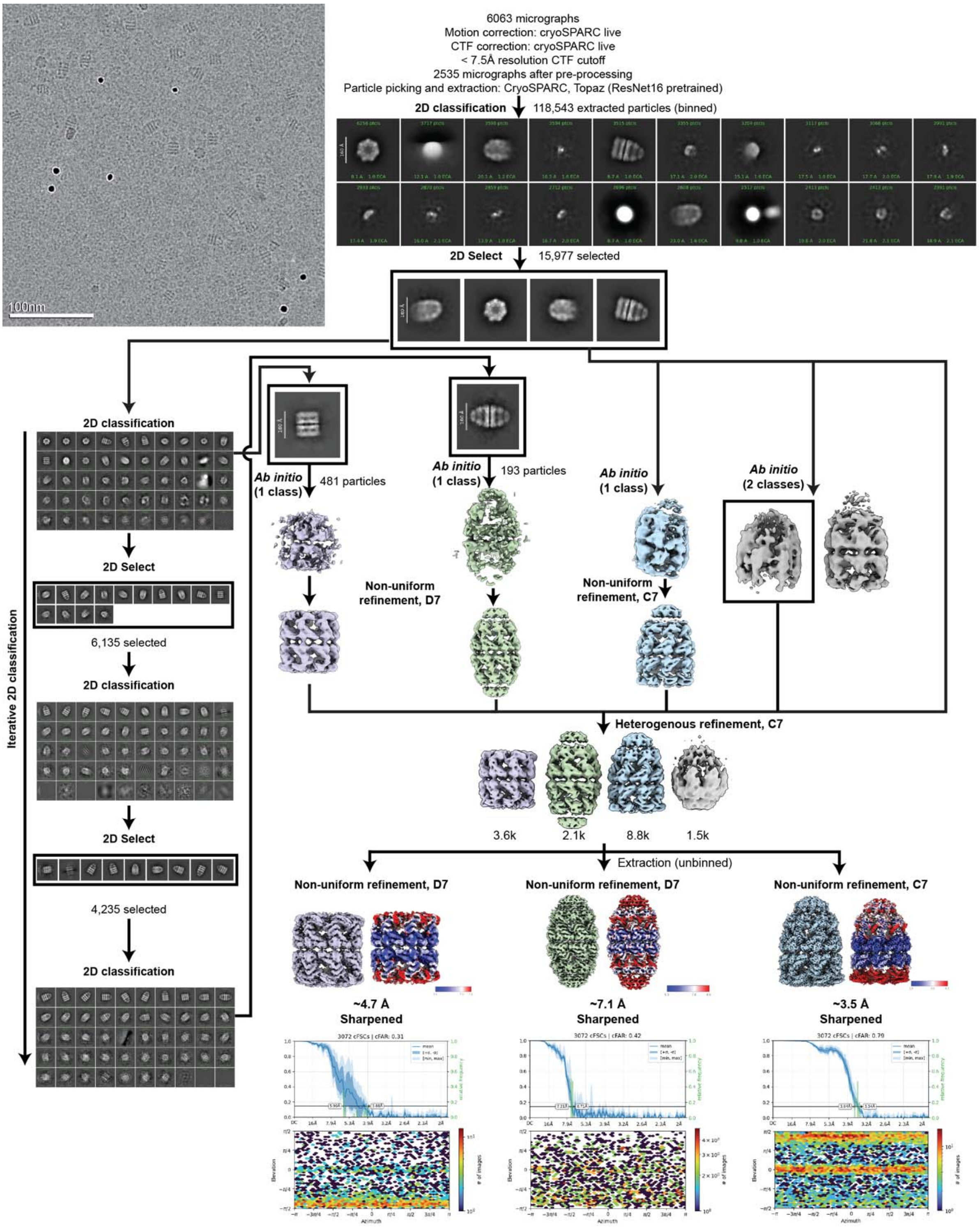

Figure S12 Processing pipeline of the 700 ms GroEL/ES + ATP dataset. Representative processing pipeline for the 700 ms GroEL + GroES + ATP dataset. Motion and CTF correction were performed in cryoSPARC Live. Micrographs were exported to cryoSPARC, and particles were picked using Topaz with a pretrained model, followed by initial 2D classification and selection of GroEL/ES complexes. Further 2D classification revealed three distinct conformational states: bullet (single-capped), barrel (uncapped), and football (double-capped). *Ab initio* reconstructions were performed independently for each conformation, and the resulting volumes were used in a heterogeneous refinement job with C7 symmetry imposed to sort particles. Sorted particles were re-extracted and refined with appropriate symmetry to yield final maps at ~3.5 Å (bullet), ~4.7 Å (barrel), and ~7.1 Å (football)

resolution. Final maps are shown next to local resolution maps. The conical FSC plot overlaid with a resolution histogram and corresponding angular distribution plot are shown below the final maps.
